# SVM and ANN Modelling Approach for the Optimization of Membrane Permeability of a Membrane Rotating Biological Contactor for Wastewater Treatment

**DOI:** 10.3390/membranes12090821

**Published:** 2022-08-23

**Authors:** Sharjeel Waqas, Noorfidza Yub Harun, Nonni Soraya Sambudi, Ushtar Arshad, Nik Abdul Hadi Md Nordin, Muhammad Roil Bilad, Anwar Ameen Hezam Saeed, Asher Ahmed Malik

**Affiliations:** 1 Chemical Engineering Department, Universiti Teknologi PETRONAS, Bandar Seri Iskandar 32610, Perak, Malaysia; 2 Department of Chemical Engineering, Universitas Pertamina, Simprug, Jakarta Selatan 12220, Indonesia; 3 Faculty of Integrated Technologies, Universiti Brunei Darussalam, Gadong BE1410, Brunei

**Keywords:** machine learning algorithm, artificial neural networks, support vector machines, membrane fouling, biological wastewater treatment

## Abstract

Membrane fouling significantly hinders the widespread application of membrane technology. In the current study, a support vector machine (SVM) and artificial neural networks (ANN) modelling approach was adopted to optimize the membrane permeability in a novel membrane rotating biological contactor (MRBC). The MRBC utilizes the disk rotation mechanism to generate a shear rate at the membrane surface to scour off the foulants. The effect of operational parameters (disk rotational speed, hydraulic retention time (HRT), and sludge retention time (SRT)) was studied on the membrane permeability. ANN and SVM are machine learning algorithms that aim to predict the model based on the trained data sets. The implementation and efficacy of machine learning and statistical approaches have been demonstrated through real-time experimental results. Feed-forward ANN with the back-propagation algorithm and SVN regression models for various kernel functions were trained to augment the membrane permeability. An overall comparison of predictive models for the test data sets reveals the model’s significance. ANN modelling with 13 hidden layers gives the highest R^2^ value of >0.99, and the SVM model with the Bayesian optimizer approach results in R^2^ values higher than 0.99. The MRBC is a promising substitute for traditional suspended growth processes, which aligns with the stipulations of ecological evolution and environmentally friendly treatment.

## 1. Introduction

The operational parameters influencing the functioning of the rotating biological contactor (RBC) bioreactor have extensively been reported [1,2,3]. Operational parameters include disk rotational speed, hydraulic retention time (HRT), sludge retention time (SRT), and carrier media type [4,5]. The selection and optimization of parameters strictly depend on the influent wastewater and effluent quality requirement. Disk rotational speed is an important parameter for acclimatizing the microorganisms and developing a full-grown biofilm to digest the organics and nutrients at the carrier surface. It is also responsible for maintaining sufficient dissolved oxygen (DO) levels inside the bioreactor to facilitate degradation [6,7,8]. The selection of SRT and loading rates relies on the wastewater strength and effluent requirements. A short SRT only facilitates carbon deduction, whereas a longer SRT results in increased sludge concentration, hindering oxygen transfer [9].

Almost 90% of the global wastewater is discharged into the mainstream without any treatment. Treating and recycling wastewater are essential for a sustainable environment and ecological atmosphere [10]. In recent years, membrane technology, in conjunction with biological treatment, has improved the effluent quality and removal efficiency [11,12].

RBC is a biological process that employs attached growth bacteria for wastewater treatment. Membrane-integrated RBC as post-treatment has shown great significance [11,13]. The performance of the RBC bioreactor depends on the operational parameters such as disk rotational speed, HRT, SRT, and microbial activity [14]. The DO is provided to the microorganism through disk rotation. Hence, disk rotational speed is an important parameter in controlling the DO levels and microbial community inside the bioreactor. The selection of appropriate loading rates (HRT and SRT) is important for optimizing the bioreactor function [15,16].

Membrane rotating biological contactor (MRBC)—a novel membrane-integrated RBC bioreactor—is a hybrid process in which a membrane is placed between two rotating disks to scour off the foulants through the generation of shear rate [17]. The disk rotation speed generates a shear rate near the membrane surface to control the fouling. Recent studies have revealed that higher HRT results in low hydraulic and organic loading rates and subsequently low viscosity and sludge concentration. A higher HRT and SRT can facilitate a higher conversion efficiency and, consequently, a higher membrane permeability [18,19,20].

In recent years, great emphasis has been given to the control of membrane fouling using artificial neural networks (ANN) and support vector machine (SVM) [21,22]. An effective way to dampen the membrane fouling is the optimization of operational parameters [23]. The ANN has been successfully applied in microfiltration/ultrafiltration to predict the system performance, the relationship of different operational parameters with membrane fouling, and the optimization of the membrane fabrication process [24,25]. Chakraborty et al. [26] predicted the membrane flux by optimizing process parameters using an ANN model for the chromium-containing aqueous solution. The predicted model is based on the Bayesian algorithm and consists of two hidden layers. The results of the ANN model are more precise than the conventional response surface methodology regression analysis. Soleimani et al. [27] studied the oily wastewater to control the membrane fouling using an ANN model. The process parameters (temperature, trans-membrane pressure, pH, and velocity) are optimized using a feed-forward ANN model with back-propagation. The predicted model results are in excellent agreement (R^2^ > 0.99) with the experimental and trained data. Rahmanian et al. [28] studied the UF treatment of wastewater by designing an ANN model. The operating parameters (pH, trans-membrane pressure, feed concentration, and electrolyte concentration) were optimized using a feed-forward ANN model. The predicted model results suggest applying ANN as an effective tool to predict complex non-linear relationships. SVM is an empirical model technique showing promising results for non-linear functions and limited data sets [29]. Meng et al. [29] applied SVM to analyze membrane fouling control and predicted complex filtration behaviors. Therefore, ANN and SVM have been applied to the current system to optimize operational parameters to generate a higher shear rate and reduce membrane fouling [30].

Disk rotational speed, HRT, and SRT are significant parameters influencing the performance of the MRBC bioreactor. The present study investigated the effect of disk rotational speed, HRT, and SRT on membrane permeability. The objective of the current study is to explore the biological performance of the RBC bioreactor treating domestic wastewater, focusing on the effect of disk rotational speed, HRT, and SRT. The focus of the study is the development of an ANN prediction model for membrane permeability. A feed-forward ANN model with back-propagation was used to predict and train the data sets for the operational parameters. This study also focuses on SVM modelling for the optimization of operating parameters. A comparison of ANN and SVM is also demonstrated through the assessment of R^2^ and various error functions.

## 2. Materials and Methods

### 2.1. Sludge Inoculation

The sludge for the bacteria cultivation was collected from the full-scale wastewater treatment plant. The sludge was allowed to acclimatize the biofilm atop the polyurethane form attached to the disks. The bioreactor was fed with constant flow wastewater during the acclimatization period. The physical observation of the biofilm was performed regularly to see any changes. The biological performance of the bioreactor was calculated after the biofilm acclimatization. 

### 2.2. Wastewater Preparation

The synthetic wastewater for the experimentation was prepared by blending leftover food (1 g/L). The prepared wastewater was left to settle for 2 h to remove the insoluble impurities. Physical treatment of the synthetic wastewater was performed by filtering through a 0.45-micron filter paper. The stock solution was diluted to match the municipal wastewater concentration. The prepared wastewater was analyzed to determine the chemical oxygen demand (COD), total nitrogen (TN), ammonium, turbidity, pH, and nitrate, as shown in Table 1.

### 2.3. Bioreactor Set-up and Operation

**Figure 1:**
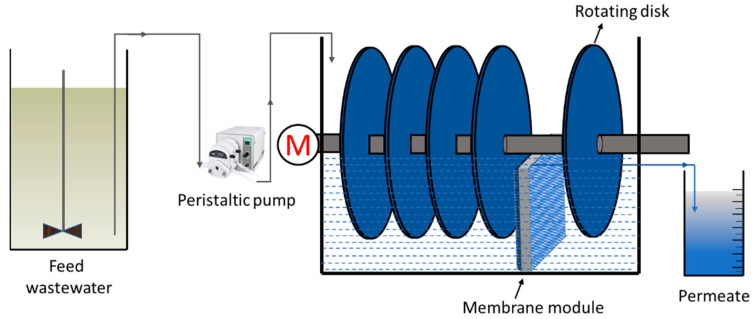
Schematic diagram of RBC-ME configuration.

The bioreactor was fabricated in-house from acrylic sheets, as shown in Figure 1. The feed wastewater tank consists of a 45 L capacity fitted with a mechanical stirrer to keep the concentration of the feed wastewater consistent. The bioreactor had 25 × 25 × 30 cm^3^ dimensions and had a working volume of 6.5 L fabricated from the acrylic sheets. The bioreactor was fitted with a stainless-steel shaft driven by a DC motor at variable speed (30–200 rpm). Five disks of 1 cm thickness and 18 cm diameter were attached to the stainless-steel shaft. The disks were covered on both sides with polyurethane foam for the inoculation of bacteria. The disks rotated inside the bioreactor at 40% disk submergence. The wastewater from the storage tank was fed continuously to the RBC bioreactor, and treated effluent was transferred to the settling tank. A mechanical stirrer continuously stirred the feed wastewater at 100 rpm to keep the concentration uniform.

For the biofilm acclimatization, the sludge was poured on the rotating disks, and the bioreactor was fed with a constant organic loading rate and HRT. No sludge was discharged during the acclimatization period. The carbonaceous bacteria acclimatize in 3–5 days, while nitrifying bacteria require 14–17 days. The biofilm was physically observed for any changes during the acclimatization period. After completing the first phase of the experiments, the bioreactor achieved a steady-state effluent concentration. After biofilm acclimatization, the system was investigated for the effect of disk rotational speed, HRT, and SRT on membrane permeability. The organic loading rates were kept constant throughout the experiments. The disk rotational speed was increased from 30 to 50 rpm with an interval increase of 5, while SRT was increased from 5 to 15 d with an interval increase of 2.5. The SRT was set by wasting a calculated amount of sludge from the bioreactor daily.

### 2.4. Machine Learning Modelling

Machine learning algorithms’ main goal is to fit the model to training data with the ultimate aim of successfully predicting unknown test data. Good training quality, on the other hand, does not necessarily correlate to consistent test results. Overfitting is a well-known example of this. Typically, an overfitted model has a modest training error but a large test error. The model has acquired an excessive number of undesirable precise information from the training data and does not fit the unknown test data. Overfitting may develop due to insufficient training processes and internal limitations, resulting in a more sensitive and complicated model. To solve the overfitting issue, an internal validation procedure called cross-validation is used [31,32].

The artificial neural network and support vector machines were used in this research to simulate the membrane permeability based on the combined influence of predictor variables, namely disk rotational speed, HRT, and SRT (Table 2). The process flow of the machine learning models employed in this study is shown in Figure 1. Following appropriate data collection, the modelling procedure was carried out using MATLAB 2020b. The trained model was chosen with the greatest accuracy achievable based on the root mean square error and R^2^.

### 2.5. Artificial Neural Network

**Figure 2:**
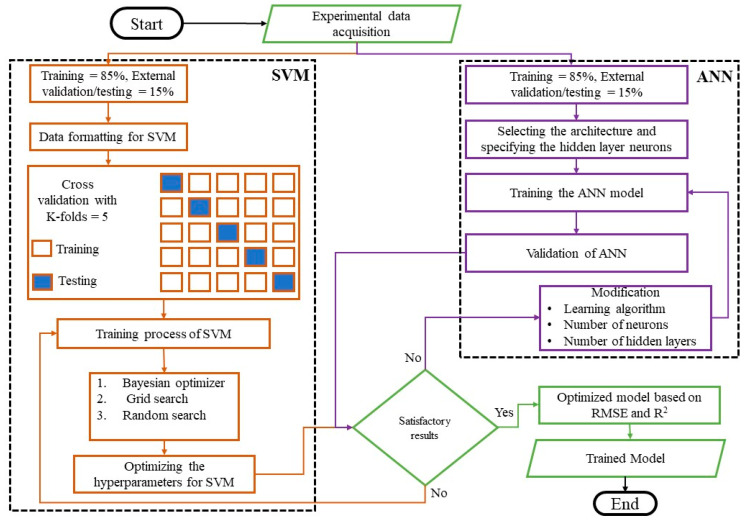
Process flow of artificial neural network and support vector machine.

Machine learning is the technology that is largely used for prediction modelling by several recent research in the field of engineering [33,34]. In regard to machine learning, ANN is specialized computational algorithms whose application is inspired by the human central nervous system. Multilayer feed-forward neural network (MLFNN) is the commonest type of ANN, extensively used in predictive modelling and analyses. It is a back-propagation learning algorithm that, in this study, is based on the Lavenberg–Marquardt model that uses the Guass–Newton approach. This typical MLFNN network consists of an input layer of neurons interconnected by weights to the succeeding hidden layers, where the input data is processed through an activation function. Finally, this processed information is transmitted to the output layer. An illustration of a typical neural network is shown in Figure 2. The number of hidden layers, the corresponding neurons, and activation functions are iteratively varied to obtain an optimized ANN model.

### 2.6. Support Vector Machine

SVM is a recent statistical machine learning technique based on an optimization algorithm invented by Wang et al. [35]. Initially, this method was only utilized for classification tasks. It has recently been developed to tackle regression problems [35]. It has been accepted as a successful strategy for QSPR investigations due to its simplicity in dealing with complicated non-linear problems, given that the outputs are real values rather than 1 or −1 [36]. This may be accomplished by mapping the non-linear characteristics of the experimental data x in a high dimensional domain with an equally efficient alternative loss function and then using linear regression in the feature space [37].

**Figure 3:**
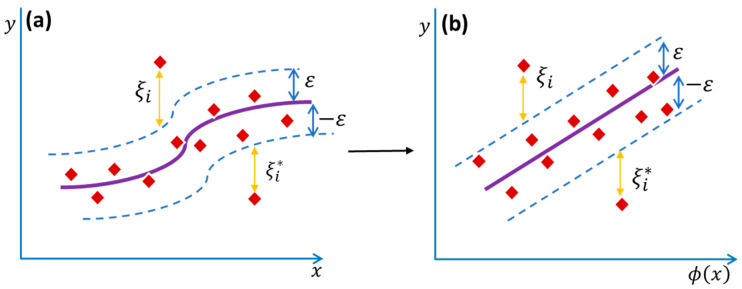
Support vector regression for non-linear response modelling from (**a**) higher dimensional domain to (**b**) lower dimensional domain.

SVM is an algorithm that is based on the principles of machine learning. SVM is based on structural risk minimization (SRM), which reduces over-fitting and increases generalization by minimizing the learning model’s projected error [38]. SVM does not provide a pre-determined structure, since the training samples’ contributions judge the training data sets’ contributions. Only chosen data samples are used for the final model development, known as “support vectors.” Figure 3 depicts the modelling process and data shifting into a chosen dimensional space. The SVM uses the objective function presented in Equation (1).

(1) $$\min\frac{1}{2}{\left\|w\right\|}^{2}+C\overset{n}{\underset{i=1}{\sum }}\left(\xi_{i}+\xi_{i}^{*}\right)$$

Given,

$$\left(w\phi \left(x_{i}\right)+b\right)-y_{i}\leq \varepsilon +\xi_{i}$$

$$y_{i}-\left(w\phi \left(x_{i}\right)+b\right)\leq \varepsilon +\xi_{i}^{*}$$

where *w* represents the direction vector, and *C* denotes the adjustment factor, which is a trade-off between training error and the flatness of the model. $\xi_{i}^{*}$ and $\xi_{i}$ are known as slack variables, and $\phi \left(x_{i}\right)$ accounts for the higher dimensional hyperspace for the input vector $x_{i}$.

There are two components to Equation (1). The first is the goal function, and the second compensates for the fitting error. To accomplish its goodness of fit, the SVM model employs the notion of minimizing of summation of errors. SVMs employ kernel functions to convert data from a lower-dimensional space to a higher-dimensional domain. The most often-utilized kernel functions are the radial basis function (RBF), linear, Gaussian, polynomial, and non-linear functions [31].

## 3. Results and Discussion

### 3.1. Artificial Neural Networks

The data are divided into three categories in the ANN modeling process: 70% training, 15% validation, and 15% testing. The MLFNN equipped with the Lavenberg–Marquardt algorithm adjusts the weights using back-propagation to reduce the error function value. This cycle is repeated until the error function reaches the minimal value and stabilizes; thus, the network is declared trained. MLFNN is not only simple but is faster in the training process and, at the same time, is capable of learning non-linear models in real-time. In this research, the sigmoid function “tansig” and the linear activation function “purlin” are used in the hidden and output layers. The “tansig” activation function is given in Equations (2) and (3) [39].

(2) $$f\left(x\right)=\frac{e^{x}-e^{-x}}{e^{x}+e^{-x}}\;$$

Given

(3) $$\;x_{j}=\overset{N}{\underset{i=1}{\sum }}w_{ij}y_{i}+b_{j}\;$$

where *x* in Equation (2) is the weighted sum of the inputs, which is calculated in terms of weights (*w*), biases (*b*), and output (*y*) according to Equation (3).

**Figure 4:**
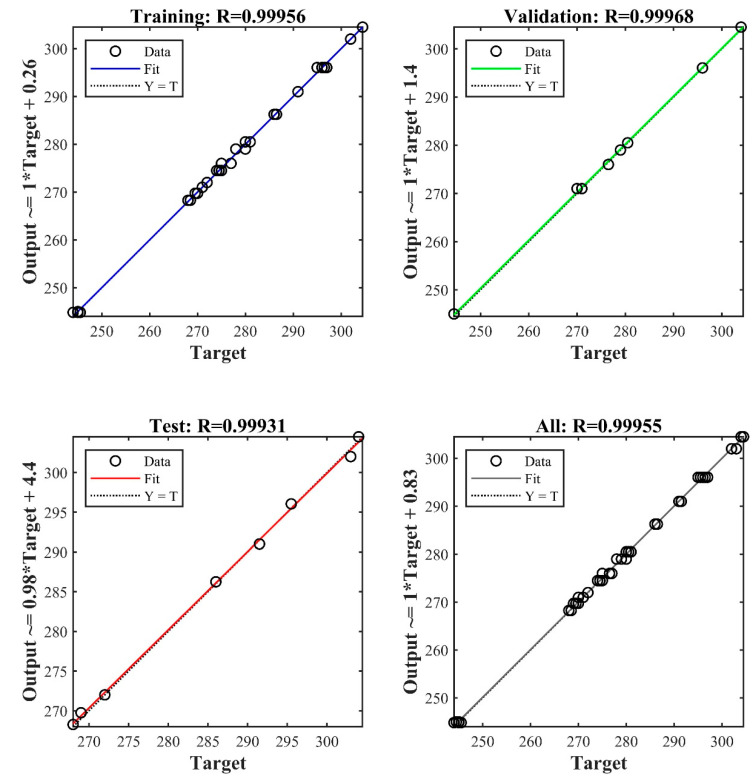
R^2^ values for trained artificial neural networks.

**Figure 5:**
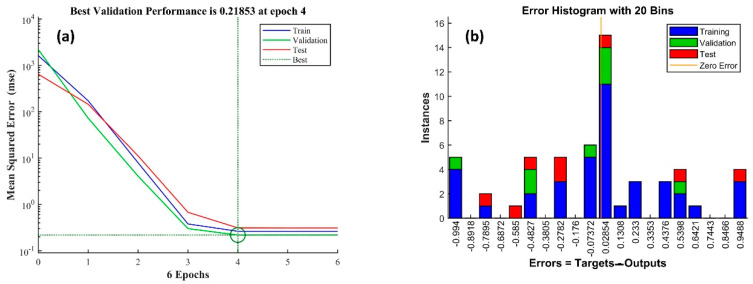
(**a**) Training performance and (**b**) error histogram of the trained network.

**Figure 6:**
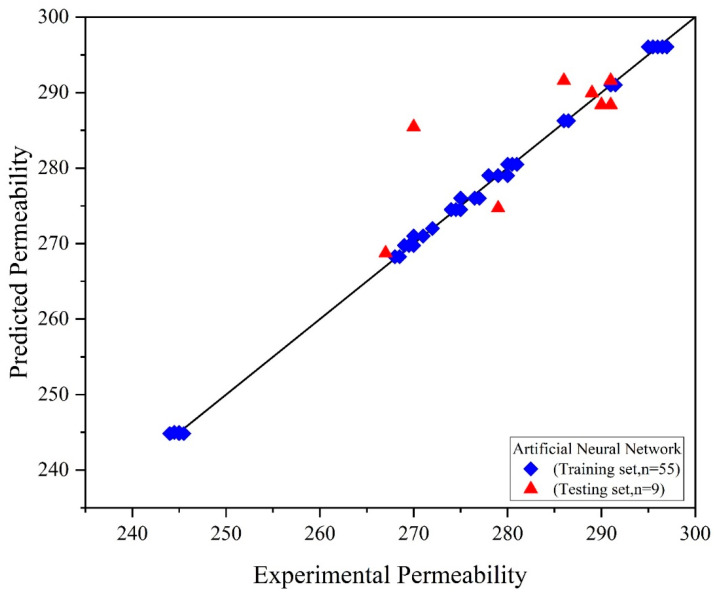
Actual vs. predicted permeability by ANN.

The most optimized network was obtained with one hidden layer containing 13 hidden neurons. The performance index R-squared given in Figure 4, and MSE values for the training testing and validation for the trained network are 0.99, 0.99, 0.99, and 0.26, 0.31, and 0.21, respectively. Error histogram and best validation performance are given in Figure 5. Figure 6 shows the comparison with training and testing performance using predictive permeability and actual permeability.

### 3.2. Support Vector Machine

**Figure 7:**
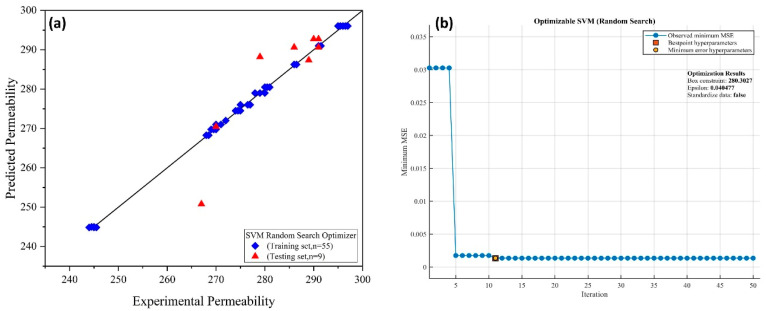
(**a**) Experimental vs. predicted membrane permeability and (**b**) training of SVM using random search optimization.

**Figure 8:**
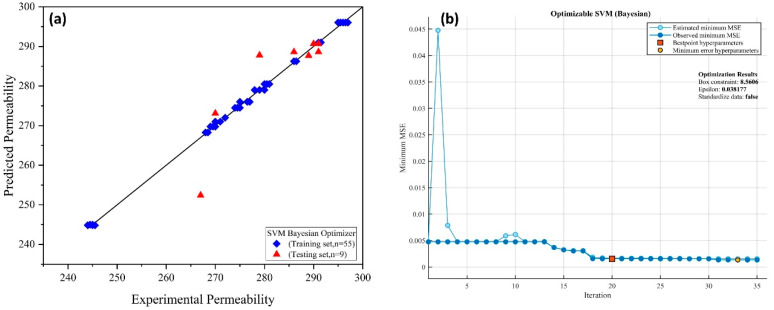
(**a**) Experimental vs. predicted membrane permeability and (**b**) training of SVM using Bayesian optimization.

**Figure 9:**
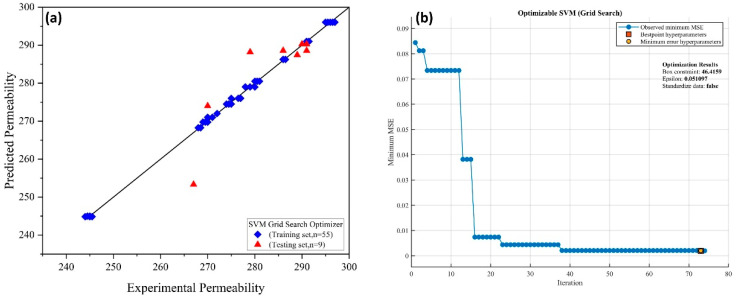
(**a**) Experimental vs. predicted membrane permeability and (**b**) training of SVM using grid search optimization.

The data in this research were separated into training and external validation/testing sets at a ratio of 85 percent and 15%, respectively. The cross-validation folds for training the SVM models were set to k-folds = 5. Three optimization techniques were used in the training process: Bayesian optimization, grid search, and random search. The hyperparameters were tuned throughout the training phase until acceptable results were achieved. Various kernel functions were tested in this research to see which was the best among them for developing a robust model. The cubic kernel function was found suitable with the best-optimized model, as shown in Equation (3) [40]. Figure 7, Figure 8 and Figure 9 show the training of SVMs with random search, Bayesian, and grid search optimizers, along with the training and testing results.

(4) $$k\left(x,y\right)={\left[\left(x\cdot y\right)+1\right]}^{3}$$

SVM has been applied to predict the membrane permeability for the operating parameters (disk rotational speed, HRT, and SRT). Previous studies also show that SVM has been applied to validate the effect of operating parameters, membrane properties, and filtrate characteristics on membrane fouling [41,42,43]. SVM modelling approach outperforms other models in terms of membrane resistance estimation in the membrane bioreactor [44]. The database of SVM training models can be used to predict the membrane fouling behavior for the unknown data sets. Thus, the non-linear relationship between the operational and output parameters (membrane fouling) results in efficient and powerful perdition SVM models compared to traditional empirical for the complex filtration processes.

### 3.3. Performance Comparison of Trained Models

The performance of the models developed through ANN and SVM were compared for the correlation coefficient (R^2^), RMSE, MBE, MAE, and NSE. The R^2^ value shows whether a linear relationship lies between the expected and observed membrane fouling values. RSME indicates the difference between the expected vs. calculated value. Table 3 shows the performance comparison of the trained modes through ANN and SVM for both the trained and unseen data sets. Table 3 shows that the ANN model with 13 nodes results in the best R^2^ value of 0.999 along with other error indexes for the trained data set. The R^2^ for the SVM Bayesian optimizer results in a 0.992 value, indicating superior performance compared to SVM grid search and SVM random search.

## 4. Conclusions

Membrane fouling dampens the application of membrane technology for wastewater treatment. The utilization of disk rotation to supply oxygen to microorganisms results in higher microbial activity and subsequently higher membrane permeability. The membrane placed in between two rotating disks results in a compact design with high removal efficiencies. In this study, the MRBC employs shear generation through disk rotation to reduce membrane fouling. The SVM and ANN modelling approach optimizes the operational parameters (disk rotational speed, HRT, and SRT). A higher value for HRT (18 h) and SRT (15 d) enables higher membrane fouling control. The SVM and ANN modelling results showed that all three operational parameters notably affect the membrane permeability. The SVM and ANN result in a higher R^2^ value (>0.99), indicating the model’s significance. The predictive model was tested for the unknown data sets, and the findings are in close agreement with the proposed model. The application of optimized and decentralized MRBC can result in a sustainable and cleaner environment.

## Figures and Tables

**Table 1:** Influent wastewater characteristics.

Contaminant	Influent
COD (mg/L)	281 ± 8.5
TN (mg/L)	2.5 ± 0.19
Ammonia (mg/L)	0.66 ± 0.03
Nitrate (mg/L)	0.49 ± 0.04
Turbidity (NTU)	14.6 ± 0.55
pH	6.28 ± 0.21

COD: chemical oxygen demand, TN: total nitrogen.

**Table 2:** Experimental Data for the Model Development.

Run #	Sr #	(A) Disk Rotational Speed (rpm)	(B) HRT (h)	(C) SRT (d)	Permeability (L/m^2^ h bar)
49	1	40	15	10	296
16	2	50	12	15	275
25	3	23.2	15	10	245
34	4	40	20	10	302
8	5	30	18	5	272
51	6	40	15	10	296
43	7	40	15	10	295
18	8	50	12	15	274
44	9	40	15	10	296.5
33	10	40	9.95	10	291
5	11	50	12	5	269
36	12	40	20	10	303
38	13	40	15	1.6	286
35	14	40	20	10	302
10	15	50	18	5	277
19	16	30	18	15	278
48	17	40	15	10	296
41	18	40	15	18.4	304
39	19	40	15	1.6	286
14	20	30	12	15	270
30	21	56.8	15	10	245
47	22	40	15	10	295
24	23	50	18	15	281
23	24	50	18	15	280.5
28	25	56.8	15	10	244
9	26	30	18	5	272
53	27	40	15	10	296.5
11	28	50	18	5	276.5
1	29	30	12	5	268
26	30	23.2	15	10	244.5
32	31	40	9.95	10	291.5
6	32	50	12	5	270
20	33	30	18	15	279
46	34	40	15	10	297
15	35	30	12	15	271
55	36	40	15	10	296
50	37	40	15	10	296.5
22	38	50	18	15	280
4	39	50	12	5	269.5
3	40	30	12	5	268.5
2	41	30	12	5	268
45	42	40	15	10	296
17	43	50	12	15	274.5
42	44	40	15	18.4	304.5
29	45	56.8	15	10	245.5
12	46	50	18	5	275
40	47	40	15	18.4	304
54	48	40	15	10	296
31	49	40	9.95	10	291
27	50	23.2	15	10	245
13	51	30	12	15	271
37	52	40	15	1.6	286.5
52	53	40	15	10	295.5
21	54	30	18	15	280
7	55	30	18	5	272

**Table 3:** Performance comparison of trained models.

Error Index	ANN 13		SVM Bayesian Optimizer		SVM Grid Search		SVM Random Search	
	Train Data	Unseen Data	Train Data	Unseen Data	Train Data	Unseen Data	Train Data	Unseen Data
RMSE	0.514	5.80	2.141	6.014	2.343	5.883	1.803	6.602
MBE	0.044	1.636	−0.152	−0.75	−0.013	−0.58	0.258	−0.284
MAE	0.367	3.77	2	4.124	2.189	4.15	1.618	4.456
NSE	0.999	0.713	0.984	0.7	0.981	0.706	0.989	0.63
R^2^	0.999	0.74	0.992	0.798	0.983	0.793	0.989	0.805

## Data Availability

Not applicable.
